# Supplementary material for: The causal relationship between the human gut microbiota and pyogenic arthritis: a Mendelian randomization study
Source: Front Cell Infect Microbiol. 2024 Nov 26;14:1452480. doi: 10.3389/fcimb.2024.1452480 (PMC11629706; doi:10.3389/fcimb.2024.1452480)
Supplement: Supplementary file 1 [file DataSheet1.zip › mendelian test/ebi-a-GCST90027797.csv_leave_one_out.pdf]

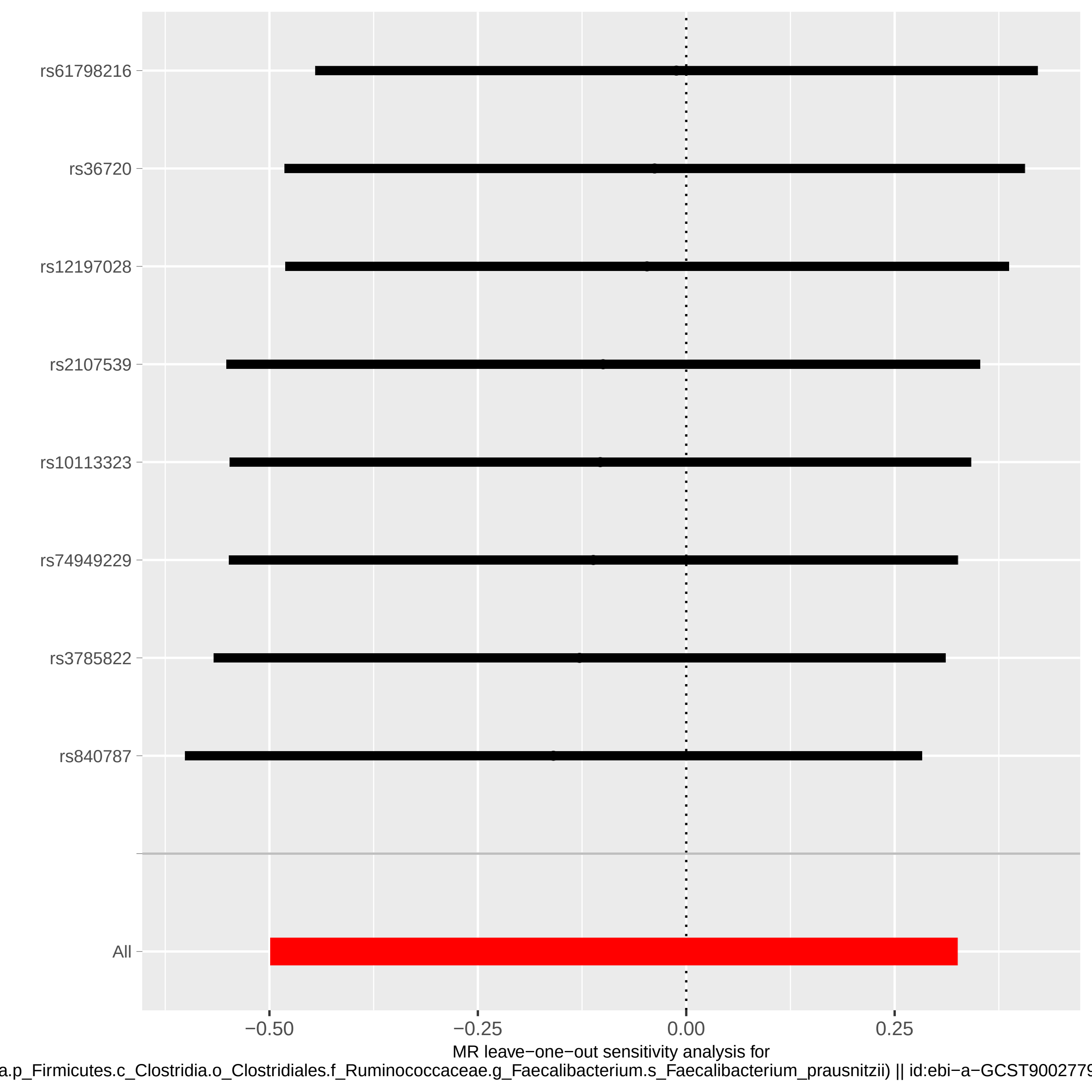

rs61798216
rs36720
rs12197028
rs2107539
rs10113323
rs74949229
rs3785822
rs840787
All
−0.50
−0.25
0.00
0.25
MR leave−one−out sensitivity analysis for
a.p_Firmicutes.c_Clostridia.o_Clostridiales.f_Ruminococcaceae.g_Faecalibacterium.s_Faecalibacterium_prausnitzii) || id:ebi−a−GCST900277
